# RETRACTED: Alhakamy et al. Development and Optimization of Luliconazole Spanlastics to Augment the Antifungal Activity against *Candida albicans*. *Pharmaceutics* 2021, *13*, 977

**DOI:** 10.3390/pharmaceutics16010150

**Published:** 2024-01-22

**Authors:** Nabil A. Alhakamy, Mohammed W. Al-Rabia, Shadab Md, Alaa Sirwi, Selwan Saud Khayat, Sahar Saad AlOtaibi, Raghad Abkar Hakami, Hadeel Al Sadoun, Basmah Medhat Eldakhakhny, Wesam H. Abdulaal, Hibah M. Aldawsari, Shaimaa M. Badr-Eldin, Mahmoud A. Elfaky

**Affiliations:** 1 Department of Pharmaceutics, Faculty of Pharmacy, King Abdulaziz University, Jeddah 21589, Saudi Arabia; nalhakamy@kau.edu.sa (N.A.A.); shaque@kau.edu.sa (S.M.); selwankhayat@gmail.com (S.S.K.); otaibisah@gmail.com (S.S.A.); raghad120012@gmail.com (R.A.H.); haldosari@kau.edu.sa (H.M.A.); 2 Advanced Drug Delivery Research Group, Faculty of Pharmacy, King Abdulaziz University, Jeddah 21589, Saudi Arabia; 3 Center of Excellence for Drug Research and Pharmaceutical Industries, King Abdulaziz University, Jeddah 21589, Saudi Arabia; 4 Department of Medical Microbiology and Parasitology, Faculty of Medicine, King Abdulaziz University, Jeddah 21589, Saudi Arabia; mwalrabia@kau.edu.sa; 5 Department of Natural Products and Alternative, Medicine, Faculty of Pharmacy, King Abdulaziz University, Jeddah 21589, Saudi Arabia; asirwi@kau.edu.sa (A.S.); melfaky@kau.edu.sa (M.A.E.); 6 Department of Medical Laboratory Technology, Faculty of Applied Medical Sciences, King Fahd Medical Research Center, King Abdulaziz University, Jeddah 21589, Saudi Arabia; hsadoun@kau.edu.sa; 7 Department of Clinical Biochemistry, Faculty of Medicine, King Abdulaziz University, Jeddah 21589, Saudi Arabia; beldakhakhny@kau.edu.sa; 8 Department of Biochemistry, Faculty of Science, Cancer and Mutagenesis Unit, King Fahd Medical Research Center, King Abdulaziz University, Jeddah 21589, Saudi Arabia; whabdulaal@kau.edu.sa; 9 Department of Pharmaceutics and Industrial Pharmacy, Faculty of Pharmacy, Cairo University, Cairo 11562, Egypt

The journal retracts the article, “Development and Optimization of Luliconazole Spanlastics to Augment the Antifungal Activity against *Candida albicans*” [1] cited above.

Following publication, concerns were brought to the attention of the publisher regarding the duplication of images.

Adhering to our complaint’s procedure, an investigation was conducted, which confirmed the duplication of Figure 3 in [1] with Figure 2B of [2].

While the authors fully cooperated with the Editorial Office during the investigation, they were unable to satisfactorily explain the overlap of figures and meet the required quality standards of raw images in order to consider a correction as per the journals original image requirements policy (https://www.mdpi.com/journal/pharmaceutics/instruc-tions#oriimages). As a result, the Editorial Board and Editor-in-Chief were unable to confirm the reliability of the findings and subsequently decided to retract this paper.

This retraction was approved by the Editor-in-Chief of the journal *Pharmaceutics*.

The authors did not agree to this retraction.
